# Defining and identifying laboratory literacy as a component of health literacy: An assessment of existing health literacy tools

**DOI:** 10.1016/j.acpath.2023.100096

**Published:** 2023-11-02

**Authors:** Jordan Franco, Nancy S. Morris, Mark K. Fung

**Affiliations:** a Robert Larner, M.D., College of Medicine at the University of Vermont, Burlington, VT, USA; b Tan Chingfen Graduate School of Nursing, UMass Chan Medical School, Worcester, MA, USA; c Department of Pathology and Laboratory Medicine, University of Vermont Medical Center, Burlington, VT, USA

**Keywords:** Health literacy, Laboratory literacy, Laboratory results, Laboratory testing

## Abstract

Health literacy has been defined and studied as an important component of a patient's ability to understand and obtain appropriate healthcare. However, a laboratory component of health literacy, as it pertains to the understanding of laboratory tests and their results, has not been previously defined. An analysis of readily available health literacy tools was conducted to determine laboratory testing-specific content representation. One hundred and four health literacy tools from a publicly available database were analyzed. Many of the health literacy tools were found to be lacking items related to laboratory testing. Of the health literacy tools that did contain a laboratory component, they were categorized pertaining to the laboratory test/testing content. Emerging from this process, eight competencies were identified that encompassed the entire range of laboratory-related aspects of health literacy. We propose that these eight competencies form the basis of a set of competencies needed for one to access, interpret, and act on laboratory results–a capacity we are referring to as “laboratory literacy.”

## Introduction

Health literacy can be broadly defined as the capacity to obtain, comprehend, and apply health-related information. Low health literacy is a determinant of health and is correlated with key outcomes, such as lower utilization of preventive services, diminished adherence to recommended medical regimes, and overall poor health outcomes.^1^ In 1974, the term “health literacy” was first coined in the scientific literature, and since the early 1990s, researchers have been investigating the field of health literacy.^2^ Fundamental to understanding health literacy is the ability to measure the concept. Numerous health literacy tools have been developed and validated to accurately gauge a patient's depth of understanding.^3^ Some of the most widely used tools can be considered “general purpose,” meaning they aim to be applicable across a range of patients and clinical contexts. Others are targeted more narrowly at assessing health literacy in specific patient populations, such as in the context of a chronic disease like diabetes or cancer. Many measures seek to gauge conceptual health literacy through the measurement of medically contextualized indicators of competency, such as reading comprehension, numeracy, and general knowledge.^4^

When considering the objectives of these tools and how they are being used by researchers and healthcare providers to drive improvements in patient outcomes, the study of a patient's understanding of laboratory testing and subsequent laboratory results would seem to be an important component of health literacy. Laboratory tests were ordered or provided in 29% of all office-based physician visits in 2016,^5^ and a recent study analyzing 3.2 billion primary care visits from 2008 to 2015 demonstrated a national decline in the number of primary care visits dedicated to reviewing laboratory results.^6^

Patients have self-reported difficulty understanding the laboratory data accessed through their electronic health portal, specifically as it pertains to the significance of their results.^7^^,^^8^ It has also been observed that patients can struggle with numeracy,^9^ the ability to understand or work with numbers, which is a key component of health literacy (National Center for Educational Statistics, n.d.).^10^ This can pose a challenge for the patient who is independently reviewing their own laboratory results, as numbers are the cornerstone of laboratory test information. Numbers are often the totality of a reported test value, and numbers form the basis for the reported “high” or “low” indicators with respect to reference ranges.

It stands to reason that a patient who misinterprets their laboratory results may experience unnecessary anxiety. If they falsely believe their results to be sufficient for good health in their situation, they may choose to make decisions regarding their health maintenance that are counterproductive. For this reason, we believe it is worth exploring the laboratory component of health literacy. Many patient populations have been characterized on the basis of measured health literacy, using tools such as the ones previously described.^2^, ^3^, ^4^ The purpose of this study was to examine existing health literacy tools to determine if they assess the contextual knowledge and skills needed to access, interpret, and act on laboratory results—a capacity we are referring to as laboratory literacy–and to define laboratory literacy as a component of health literacy.

## Material and methods

We identified our pool of health literacy tools by using *Health Literacy Tool Shed*, an online database of health literacy measures that is maintained in partnership by Boston University, CommunicateHealth Inc., and Research Triangle Institute (RTI) International.^11^ The inclusion criteria for health literacy tools listed in this database include tools, measures, and items that meet the requirement of measuring an individual's health literacy and having a published design and validation study in a peer-reviewed journal.^12^ The exclusion criteria excluded health literacy tools designed to assess provider communication, healthcare system complexity, or health literacy tools designed to assess individual components of organizations or related materials.^12^

This route was chosen for the efficiency of using a pre-constructed database. The *Health Literacy Tool Shed*, in addition to containing relevant and previously aggregated health literacy tools, provides references to each tool's published validation paper. It also provides details on the specifics of the tools including information regarding validation sample size, validated administration modes, and instrument language. It also allows one to filter the database by one or more identifiers of interest.

Using the *Find Measures* search feature of the *Health Literacy Tool Shed* database, we applied the filter function to filter all entries of health literacy tools (totaling 217 at the time of study in June 2022) to include only the ones that are in the English language. This returned 104 entries, from which we accessed the published validation study for each. One tool was removed due to being in Spanish and having been erroneously included in the filtered database results. Also removed were four database entries that were duplications or that were validations and expansions of a previously listed tool in the database results.

**Fig. 1:**
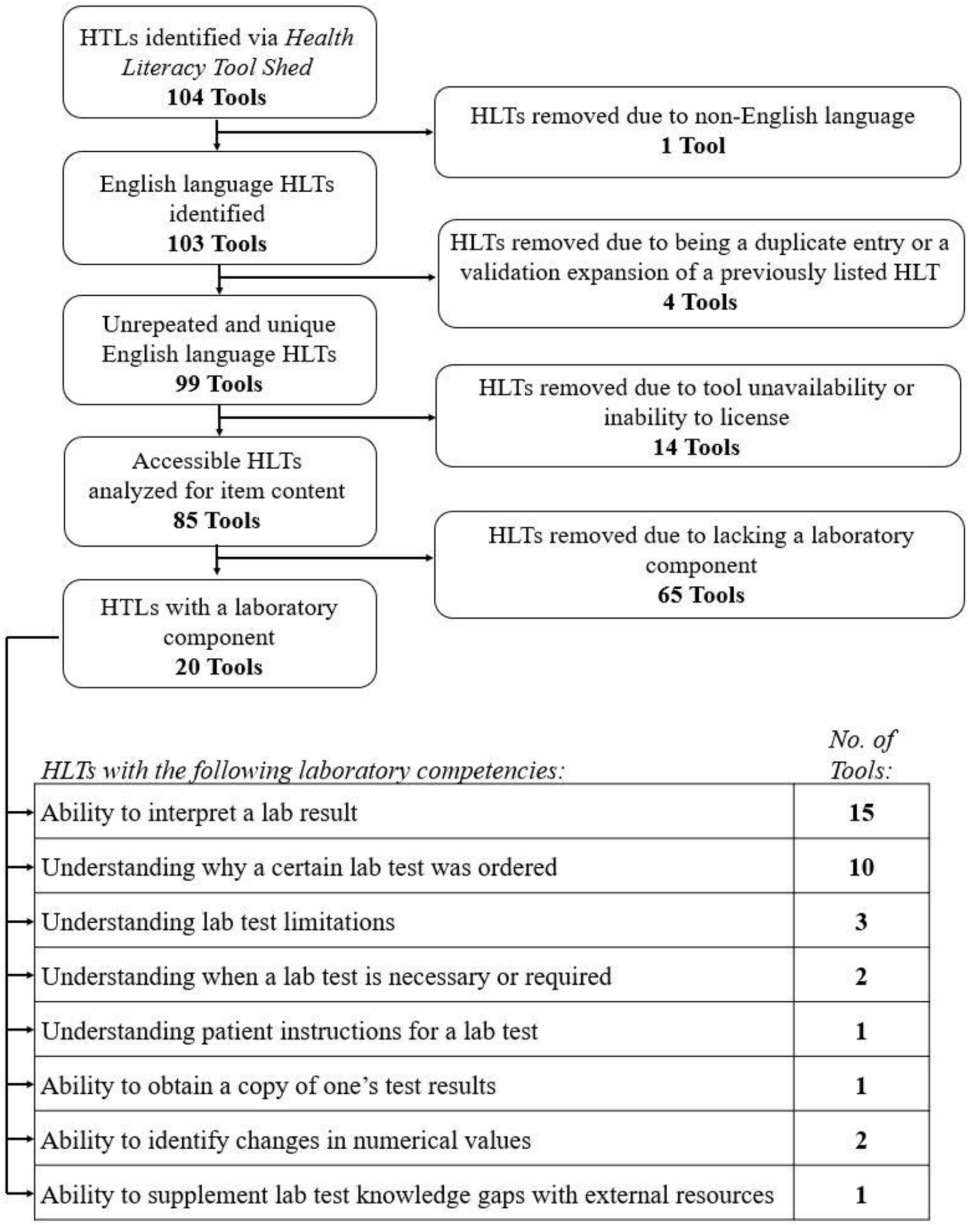
Decisions made in the health literacy tool review, along with the resulting laboratory competencies that emerged after identifying and analyzing those found to contain a laboratory component. As shown in the right column (which depicts the number of tools containing that competency), most health literacy tools with a laboratory component included one or more questions addressing multiple competencies.

This left a pool of 99 health literacy tools, many of which were readily available for viewing purposes. There were 14, however, that were inaccessible. In many instances, this was due to the tool or instrument being proprietary and requiring licensing. In all instances, an attempt was made to contact the respective author through email for viewing access, and a period of one month was allowed for a response before removing that health literacy tool from the review. The remaining 85 were accessed, and we applied the following criteria in their analysis (see Fig. 1, and Supplemental Table 1). We ascertained whether any items on the instrument contained a “laboratory component,” as defined as a component of the item that was pertinent to and addressed competencies or knowledge involved in accessing, interpreting, or acting on laboratory results. Such items were flagged and set aside as containing a laboratory component.

Only laboratory testing was considered. Clinical testing such as blood pressure readings, spirometry readings, or body mass index measurements, was not considered to involve a laboratory component. At-home blood glucose testing was considered to be a laboratory component. Assessments that were primarily oral (for example, an item testing the ability of a respondent to correctly pronounce the word “laboratory”) were not included. We made the decision that recognition and reading of more common terms would not help us know if a person could read and understand laboratory terms they likely are not familiar with. If a health literacy tool contained untested ancillary information pertaining to lab work (for example, a reading passage detailing a health condition that mentions routine blood testing but then ignores that information when asking comprehension questions), it was considered not to contain a laboratory component.

After all 85 health literacy tools were analyzed, those flagged as containing a laboratory component were scrutinized and assigned competencies delineating what particular aspects of laboratory testing that item addressed. Competencies were created, and assignments were performed independently by authors JF and MF, and then reviewed together afterward for congruence. Upon independently arriving at competencies and assignments, one competency was eliminated for being redundant, and five competencies were added*.* The items flagged as containing laboratory components, along with the overall assignment scheme, were then reviewed by third author NM.

## Results

Out of the 85 health literacy tools analyzed, 20 were found to have a relevant laboratory component.^13^, ^14^, ^15^, ^16^, ^17^, ^18^, ^19^, ^20^, ^21^, ^22^, ^23^, ^24^, ^25^, ^26^, ^27^, ^28^, ^29^ These 20 identified competencies were found to fit into eight discrete competencies based on their content (Fig. 1). The majority assessed only two of these competencies: ability to interpret a lab result (n = 15) and understanding why a certain lab test was ordered (n = 10). Half (n = 10) of the health literacy tools assessed only one laboratory literacy competency, six assessed two competencies, three assessed three competencies, and one assessed five competencies (Table 1).

**Table 1:** A descriptive table of the 20 health literacy tools observed to contain a laboratory component. Detailed is information pertaining to its intended use case. “General” refers to a general-purpose health literacy tool, while more specific terms such as “diabetes” or “cancer” describe the chronic disease process that characterizes the intended patient population for that health literacy tool. Also detailed is a breakdown of how many items in each health literacy tool contain a laboratory component and what competencies of laboratory literacy those items address.

Health literacy tool abbreviation:	Health literacy tool name:	Intended population for use:	Total items:	Total laboratory items:	Laboratory literacy competencies addressed:
CHLT-6	30-Item Cancer Health Literacy Test^13^	Cancer	6	1	1
CHLT-30	6-Item Cancer Health Literacy Test^13^	Cancer	30	1	1
CMLT-R	Cancer Message Literacy Test – Reading^14^	Cancer	23	2	1,2
CMLT-L	Cancer Message Literacy Test – Listening^14^	Cancer	48	1	1,2,7
C-CLAT	Cervical Cancer Literacy Assessment Tool^15^	Cancer	16	6	2,4
CHAS	Comprehensive Health Activities Scale^16^	General	45	1	1,2
CAHPS	Consumer Assessment of Healthcare Providers and Systems^17^	General	89	4	2
DNT-14	Diabetes Numeracy Test for Adolescents^18^	Diabetes	14	1	1
DNT-15	Diabetes Numeracy Test Short Form^19^	Diabetes	15	1	1
DNT-43	Diabetes Numeracy Test^19^	Diabetes	43	2	1
HAS-A	Health Literacy Assessment Scale for Adolescents^20^	General	15	1	1,2
Health LiTT	Health Literacy Assessment using Talking Touchscreen Technology^21^	General	82	6	1,2,5,6,7
HELIA	Health Literacy Instrument for Adults^22^	General	33	3	3,8
HLSI	Health Literacy Skills Instrument^23^	General	25	3	1
HLSI-10	Health Literacy Skills Instrument - Short Form^24^	General	10	1	1
HIV-HL	HIV-Related Health Literacy Scale^25^	HIV	25	1	2
K-TUT	Kidney Transplant Understanding Tool^26^	Kidney Disease	69	3	2,4
S-NUMi	Numeracy Understanding in Medicine Instrument (Short version)^27^	General	8	2	1,2,5
NUMi	Numeracy Understanding in Medicine Instrument^28^	General	20	2	1,2,5
WELLS	Water Environmental Literacy Level Scale^29^	Environmental Health	6	6	1

1 = Interpretation of a lab result.

2 = Understanding why a certain lab test was ordered.

3 = Understanding patient instructions for a lab test.

4 = Understanding when a lab test is necessary or required.

5 = Understanding lab test limitations.

6 = Ability to obtain a copy of one's test results.

7 = Ability to identify changes in numerical values.

8 = Ability to supplement lab test knowledge gaps with external resources.

## Discussion

**Fig. 2:**
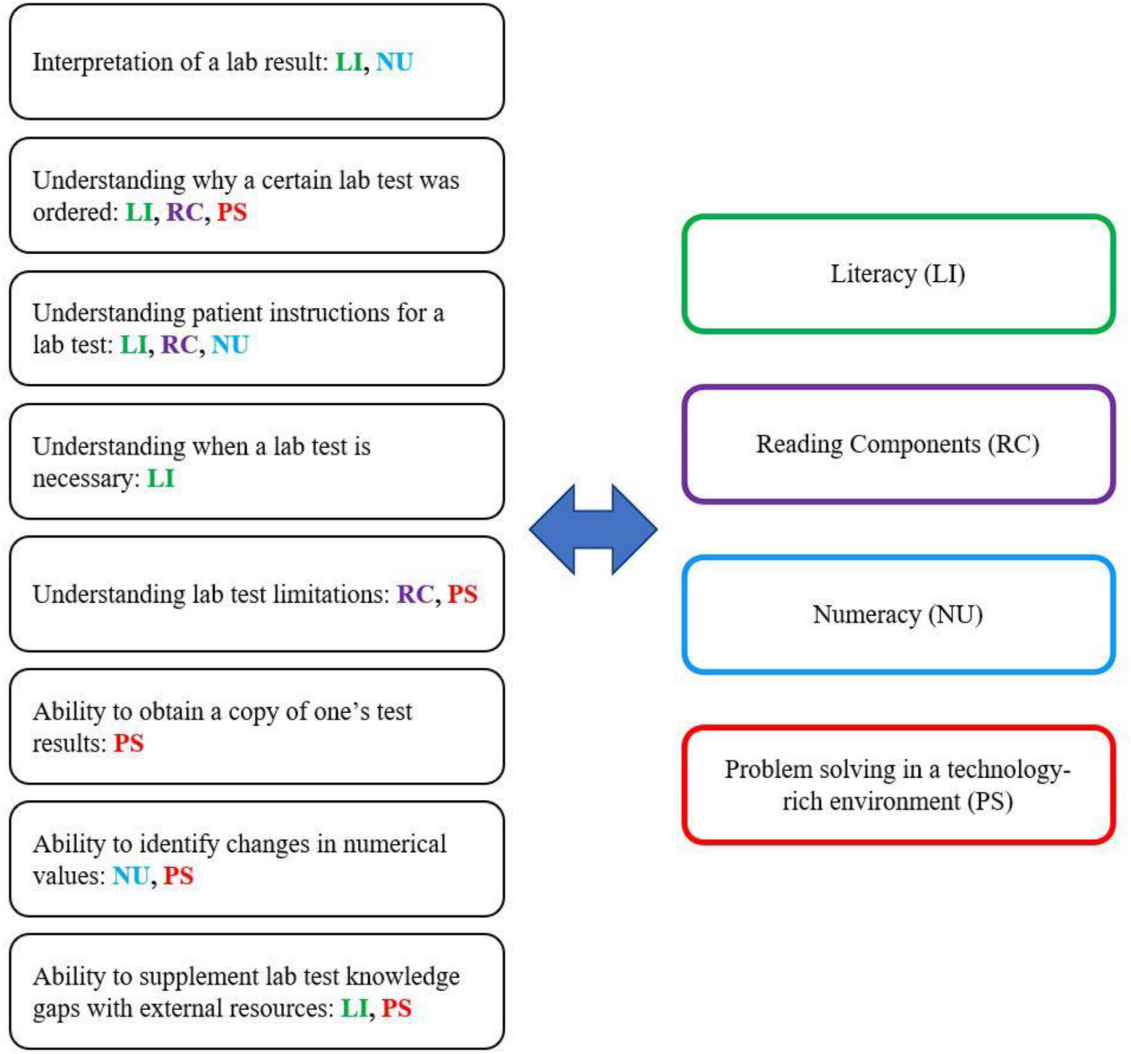
Crosswalk of emergent laboratory literacy competencies to U.S. Program for the International Assessment of Adult Competencies (PIAAC) domains.

In proposing models of health literacy, the literature discusses the following domains: fundamental literacy, scientific literacy, civic literacy, and cultural literacy^30^ and describes four competency domains, including literacy, reading components, numeracy, and problem solving in a technology-rich environment. The latter is a defined set of competencies by the *Programme for the International Assessment of Adult Competencies (*PIAAC), which is established from an internationally-recurring, cross-culture survey of adult cognitive skills.^31^ After analyzing the items of the health literacy tools containing a laboratory component in this review, the competencies they addressed were found to fit into eight discrete competencies (Fig. 1). As these emergent competencies align with the aforementioned PIAAC competencies (Fig. 2), we are proposing that they delineate associated competencies regarding laboratory literacy.

Out of the 20 health literacy tools with a laboratory component, competency 1, the *ability to interpret a lab result*, was the competency most tested. An example was found in the Cancer Health Literacy Tool (CHLT-30), a computer-based cancer health literacy test validated for use in adults aged 18–64 years old.^13^ It included an item that presents a reference range for normal hemoglobin levels and then asks whether the hemoglobin result value of a hypothetical patient is normal using a Yes/No answer format. The least tested competencies included competencies 3, 6, and 8. An example of a competency 3 test items can be found in the Health Literacy Tool for Adults (HELIA), a paper- and pencil-based general health literacy test validated for use in adults aged 18–64 years old.^22^ It asks the respondent to self-report their ability to understand written information before laboratory testing. Also in the Health Literacy Tool for Adults (HELIA) was an example of laboratory literacy competency 8, *the ability to supplement lab test knowledge gaps with external resources*, in the form of asking the respondent to self-report their ability to find health information regarding high lipid levels.

Laboratory competency 4, *understanding when a lab test is necessary*, was only tested in 2 of the 20 health literacy tools found to contain a laboratory component. The Cervical Cancer Literacy Assessment Tool (C-CLAT), a paper and pencil-based cervical cancer health literacy tool validated for use in adults aged 18–64 years old,^15^ had several items addressing this competency, asking respondents what age Pap test screening should begin and asking if an abnormal Pap test result warrants the discontinuation of regular Pap testing. The Kidney Transplant Understanding Tool (K-TUT), a mailed survey-based kidney disease health literacy tool validated for use in adults aged 18–64 years old,^26^ had several questions regarding competency 4, including one that asked about the importance of regular blood testing and one that asked about the necessity of monthly blood testing for someone with a functioning kidney transplant. Both the Kidney Transplant Understanding Tool (K-TUT) and the Cervical Cancer Literacy Assessment Tool (C-CLAT) were designed to measure health literacy in patient populations with chronic health diseases, so it makes sense that some of their instrument items were dedicated to addressing the recurring nature of laboratory testing required to monitor disease or treatment progress. It is worth noting that there were other cancer health literacy tools that did not test for competency 4, yet still tested other competencies of laboratory literacy; and there were also cancer health literacy tools that contained no components of laboratory literacy at all, leaving all competencies untested.

In the assessment of 85 health literacy tools, we came across several worth mentioning explicitly, which contained either unique test items or items testing many of the above-mentioned laboratory literacy competencies.

The 8-item *Numeracy Understanding in Medicine Instrument Short Version* (S-NUMi)^27^ was the only measure containing a test item observed to address the concept of false negative or false positive test results. This is interesting because such an item expands across multiple laboratory literacy competencies; it addresses competencies 1 and 5 (*interpretation of a lab result* and *understanding lab test limitations*, respectively) simultaneously. This type of test item has the potential to shed light on how a patient perceives the validity of their laboratory test result.

The 23-item *Cancer Message Literacy Test – Reading* (CMLT-R)^14^ contained two items pertaining to laboratory literacy, in both instances asking about Prostate-Specific Antigen (PSA) screening tests regarding a preceding passage that provided detailed information on PSA tests. Both questions addressed competencies 1 and 2 (*interpretation of a lab result* and understanding why a lab test was ordered, respectively), yet both were worded in such a way as to be multi-step questions, requiring the retention of multiple points of information detailed in the passage and then the use of them to answer a single question. For example, one question required the respondent to have retained from the passage information regarding what a PSA test is and the information needed to understand that a high value is a bad result. Such a question required the retention of information, which was not as explicitly encountered in other health literacy tools.

The 82-item *Health Literacy Assessment using Talking Touchscreen Technology* (Health LiTT),^21^ tested by far the largest number of laboratory literacy competencies out of any of the health literacy tools we reviewed. Through six items dedicated to these topics, laboratory literacy competencies 1, 2, 5, 6, and 7 were tested using a computer-based survey. A point of discussion worth mentioning is that this tool tested the ability of a respondent to understand the importance of being able to obtain a copy of their test results, which was a consideration unique among all the health literacy tools we reviewed.

A limitation of this study is our use of the *Health Literacy Tool Shed* database as our only source of health literacy tools. Although updates to this database are ongoing and occur on a regular basis,^11^ there's a possibility that relevant health literacy tools exist in the published literature that are not included in this database.

Some health literacy tools were not able to be accessed in full in the way that they would be presented to a respondent. For example, in one instance, we were only able to access a descriptive table in the Comprehensive Health Activities Scale (CHAS) paper that indicated a laboratory component for an item described as “calculate and interpret numeric information from a chart listing 7 days of recorded blood sugar levels before and after meals for a diabetic patient.”^16^ We flagged this item as pertaining to competency 1, *interpreting a lab result*. We would have preferred to have viewed the entire health literacy tool in its finalized form to properly view the test item. It is plausible that the exact wording of the item on the instrument, as presented to the respondent, could change the overall assignment of that item to having one or more additional competencies. Also, it is a possibility that other important lab literacy competencies exist but were not identified in these measures.

## Conclusion

Many health literacy tools lack components that specifically address concepts necessary for laboratory literacy. The development process of future health literacy tools should include careful consideration as to the value of incorporating laboratory literacy into their design as a distinct domain within health literacy. We believe further studies surrounding the topic of laboratory literacy are warranted, and many can be envisioned that address a host of interesting research questions. We would suggest an investigation into how the relevant competencies of laboratory literacy might change in importance and utility in the context of one disease compared to another. We would also suggest exploring if competency 8*, Ability to supplement lab test knowledge gaps with external resources,* has the potential to negate the necessity of the other competencies as a higher-order measure of laboratory literacy that incorporates other competencies of accessing, interpreting, and applying information, or perhaps as a high-level competency that builds upon these other competencies. It is an interesting proposition that having proficiency in acquiring external information to supplement their understanding might offset the health literacy effects of deficits in other competencies.

Additionally, an understanding of laboratory literacy among healthcare providers is also an important area of future investigation. Most, if not all, of the competencies identified for laboratory literacy would appear to be applicable to both patients and providers. Analogous to the challenges facing patients, an expanding array of diagnostic assays may pose challenges to providers in each of the competencies we have identified. Studies of the broader topic of health literacy among providers are relatively new, with only a single recent study suggesting gaps in health literacy awareness and limited use of guidelines to communicate more effectively with patients.^32^

When considering the impact that this field of research can have on the patient level, the provider level, the health system level, and the national level, we believe that this is a worthy area of further study. This field of inquiry into the relationship between laboratory testing and health literacy has the potential to produce knowledge that can be used to improve health literacy assessments, healthcare delivery, and ultimately to help reduce healthcare disparities and improve patient outcomes.

## Declaration of competing interest

The authors declare that they have no known competing financial interests or personal relationships that could have appeared to influence the work reported in this paper.

## Funding

The article processing fee for this article was funded by an Open Access Award given by the Society of ‘67, which supports the mission of the 10.13039/100016205Association of Pathology Chairs to produce the next generation of outstanding investigators and educational scholars in the field of pathology. This award helps to promote the publication of high-quality original scholarship in *Academic Pathology* by authors at an early stage of academic development.

This study was supported in part by the 10.13039/100010941University of Vermont Roy and Lorraine Korson Green and Gold Professorship and the 10.13039/100010941University of Vermont Larner College of Medicine Summer Medical Research Fund.
