# Supplementary material for: Defining and identifying laboratory literacy as a component of health literacy: An assessment of existing health literacy tools
Source: Acad Pathol. 2023 Nov 2;10(4):100096. doi: 10.1016/j.acpath.2023.100096 (PMC10641569; doi:10.1016/j.acpath.2023.100096)
Supplement: Multimedia component 1 [file mmc1.docx]

**Supplemental Table 1.** A listing of the 99 unique English language health literacy tools considered in this review.

| Abbreviation | Name | Supplemental Reference |
| --- | --- | --- |
| CHLT-30 | 30-Item Cancer Health Literacy Test | Dumenci et al.^1^ |
| CHLT-6 | 6-Item Cancer Health Literacy Test | Dumenci et al.^1^ |
| S-TOFHLA | Abbreviated version of the Test of Functional Health Literacy in Adults | Baker et al.^2^ |
| AAHLS | All Aspects of Health Literacy Scale | Chinn et al.^3^ |
| A-REALM | Arthritis-Adapted Rapid Estimate of Adult Literacy in Medicine | Swearingen et al.^4^ |
|  | Berlin Numeracy Test | Cokely et al.^5^ |
| B-CLAT | Breast Cancer Literacy Assessment Tool | Williams et al.^6^ |
| BEHKA-HIV | Brief Estimate of Health Knowledge and Action - HIV Version | Osborn et al.^7^ |
|  | Brief Health Literacy Screener (Chew items) | Chew et al.^8^ |
| BRIEF | Brief Health Literacy Screening Tool | Haun et al.^9^ |
|  | Calgary Charter on Health Literacy Scale | Pleasant et al.^10^ |
| CMLT-R | Cancer Message Literacy Test - Reading - CMLT-Reading | Mazor et al.^11^ |
| CMLT-L | Cancer Message Literacy Test - Listening - CMLT-Listening | Mazor et al.^11^ |
| C-CLAT | Cervical Cancer Literacy Assessment Tool | Williams and Templin^12^ |
|  | Claim Evaluation Tools Database | Austvoll-Dahlgren et al.^13^ |
| HLS/SNS | Composite Health Literacy Scale and Subjective Numeracy Scale - HLS/SNS | Luo et al.^14^ |
|  | Comprehension of 50 Medical Terms | Samara et al.^15^ |
| CHAS | Comprehensive Health Activities Scale | Curtis et al.^16^ |
| CAHPS | Consumer Assessment of Healthcare Providers and Systems | Weidmer et al.^17^ |
|  | Critical Nutrition Literacy Instrument | Guttersrud et al.^18^ |
| DAHL | Demographic Assessment for Health Literacy | Hanchate et al.^19^ |
| REALM-Teen | Rapid Estimate of Adolescent Literacy in Medicine | Davis et al.^20^ |
| DNT-14 | Diabetes Numeracy Test (adolescents) | Mulvaney et al.^21^ |
| DNT-15 | Diabetes Numeracy Test Short Form | Huizinga et al.^22^ |
|  | Diabetes Numeracy Test | Huizinga et al.^22^ |
| EMAHL-13 | Eastern-Middle Eastern Adult Health Literacy 13 Point Questionnaire | Kayser et al.^23^ |
| eHLQ | eHealth Literacy Questionnaire | Kayser et al.^23^ |
| eHEALS | eHealth Literacy Scale | Norman and Skinner^24^ |
| HLS-EU-Q6 | European Health Literacy Survey | Rouquette et al.^25^ |
| FLBC | Evaluation Tool Development for Food Literacy Programs | Begley et al.^26^ |
| FNLIT | Food and Nutrition Literacy | Doustmohammadian et al.^27^ |
| FLLANK | Food Label Literacy for Applied Nutrition Knowledge Questionnaire | Reynolds et al.^28^ |
| FLIGHT | Fostering Literacy for Good Health Today | Ownby et al.^29^ |
| FHLT | Functional Health Literacy Test | Zhang et al.^30^ |
| GHNT-21 | General Health Numeracy Test | Osborn et al.^31^ |
| GHNT-6 | General Health Numeracy Test | Osborn et al.^31^ |
|  | Graph Literacy Scale | Galesic and Garcia-Retamero ^32^ |
| HALS | Health Activities Literacy Scale of NALS | Rudd et al.^33^ |
| HAS-A | Health Literacy Assessment Scale for Adolescents | Manganello et al.^34^ |
| HLAT-8 | Health Literacy Assessment Tool | Abel et al.^35^ |
| Health LiTT | Health Literacy Assessment using Talking Touchscreen Technology | Hahn et al.^36^ |
| HLSAC | Health Literacy for School-Aged Children | Paakkari et al.^37^ |
| HeLD | Health Literacy in Dentistry Scale | Jones et al.^38^ |
| HELIA | Health Literacy Instrument for Adults | Tavousi et al.^39^ |
| HeLMS | Health Literacy Management Scale | Jordan et al.^40^ |
| HELMA | Health Literacy Measure for Adolescents | Ghanbari et al.^41^ |
|  | Health Literacy Measure for High School Students | Wu et al.^42^ |
| HLCS-C | Health Literacy of Caregivers Scale - Cancer | Yuen et al.^43^ |
| HLQ | Health Literacy Questionnaire | Osborne et al.^44^ |
| HLSI | Health Literacy Skills Instrument | McCormack et al.^45^ |
| HLSI-10 | Health Literacy Skills Instrument - Short Form | Bann et al.^46^ |
| HLTS | Health Literacy Test for Singapore | Ko et al.^47^ |
| HBP-HLS | High Blood Pressure Health Literacy Scale | Kim et al.^48^ |
| HIV-HL | HIV-Related Health Literacy Scale | Ownby et al.^49^ |
| HHLAT | Hypertension Health Literacy Assessment Tool | Mafutha et al.^50^ |
| IDLS | Intellectual Disability Literacy | Scior and Furnham^51^ |
| K-TUT | Kidney Transplant Understanding Tool | Rosaasen et al.^52^ |
| LAD | Literacy Assessment for Diabetes | Nath et al.^53^ |
| MHL | Media Health Literacy Measure | Levin-Zamir et al.^54^ |
| MART | Medical Achievment Reading Test | Hanson-Divers^55^ |
| MDIT | Medical Data Interpretation Test | Schwartz et al.^56^ |
| METER | Medical Term Recognition Test | Rawson et al.^57^ |
| MedLitRxSE | Medication Literacy Assessment in Spanish & English | Sauceda et al.^58^ |
| NVS | Newest Vital Sign | Weiss et al.^59^ |
| NVS-UK | Newest Vital Sign (UK Version) | Rowlands et al.^60^ |
|  | Numeracy Scale | Lipkus et al.^61^ |
| S-NUMi | Numeracy Understanding in Medicine Instrument (Short version) | Schapira et al.^62^ |
| NUMi | Numeracy Understanding in Medicine Instrument (Short version) | Schapira et al.^63^ |
| NLit | Nutrition Health Literacy Instrument for Adults with Chronic Health Conditions | Gibbs et al.^64^ |
| NLit | Nutrition Health Literacy Instrument | Gibbs et al.^65^ |
| NLS | Nutritional Literacy Scale | Diamond^66^ |
| OHLI | Oral Health Literacy Instrument | Sabbahi et al.^67^ |
| PHLAT | Parental Health Literacy Activities Test | Kumar et al.^68^ |
| PPSI | Parenting Plus Skills Index | Ayre et al.^69^ |
| REALM-TeenS | Rapid Estimate of Adolescent Literacy in Medicine Short Form | Manganello et al.^70^ |
| REALD-99 | Rapid Estimate of Adult Literacy in Dentistry | Richman et al.^71^ |
| REALD-30 | Rapid Estimate of Adult Literacy in Dentistry 30 Short Form | Lee et al. ^72^ |
| REAL-G (short form) | Rapid Estimate of Adult Literacy in Genetics (short form) | Erby et al. ^73^ |
| REAL-G | Rapid Estimate of Adult Literacy in Genetics | Erby et al. ^73^ |
| REALM-R (short) | Rapid Estimate of Adult Literacy in Medicine (Rapid) | Bass et al. ^74^ |
| REALM | Rapid Estimate of Adult Literacy in Medicine | Davis et al. ^75^ |
| REALM-D | Rapid Estimate of Adult Literacy in Medicine and Dentistry | Atchison et al.^76^ |
| REALM-SF | Rapid Estimate of Adult Literacy in Medicine (Short form) | Arozullah et al.^77^ |
| REAL_VS | Rapid Estimate of Adult Literacy in Vascular Surgery | Wallace et al.^78^ |
| REAL_VSs | Rapid Estimate of Adult Literacy in Vascular Surgery (short form) | Wallace et al.^78^ |
| SAHL-E | Short Assessment of Health Literacy (Spanish & English) | Lee et al.^79^ |
| HLS-SF12 | Short Form Health Literacy Questionnaire for Asian Populations | Duong et al.^80^ |
| 14-item MedLitRxSE | Shortened Medication Literacy Assessment in Spanish & English | Sauceda et al.^58^ |
| SNS-3 | Shortened Subjective Numeracy Scale | McNaughton et al.^81^ |
| SILS | Single Item Literacy Screener | Morris et al.^82^ |
| SML | Smoking Media Literacy | Primack et al.^83^ |
| SNS | Subjective Numeracy Scale | Fagerlin et al.^84^ |
| HLS-CH | Swiss Health Literacy Survey | Wang et al. ^85^ |
| TOFHLA-iD | Test of Functional Health Literacy in Dentistry | Gong et al. ^86^ |
|  | The Role of Numeracy in Understanding the Benefit of Screening Mammography | Schwartz et al.^87^ |
| eHEALS | Three-Factor Structure of the eHealth Literacy Scale Among MRI and CT Outpatients | Hyde et al. ^88^ |
| TS-REALD | Two-Stage Rapid Estimate of Adult Literacy in Dentistry | Stucky et al.^89^ |
| WELLS | Water Environmental Literacy Level Scale | Irvin et al. ^90^ |
|  | Weight Literacy Scale in English and Spanish | Wang et al.^91^ |

**Supplemental References**

1. Dumenci L, Matsuyama R, Riddle DL, et al. Measurement of cancer health literacy and identification of patients with limited cancer health literacy.*J Health Commun.*2014;19 Suppl 2(0 2):205-24. doi:10.1080/10810730.2014.943377

2. Baker DW, Williams MV, Parker RM, Gazmararian JA, Nurss J. Development of a brief test to measure functional health literacy.*Patient Educ Couns.*1999;38(1):33-42. doi:10.1016/s0738-3991(98)00116-5

3. Chinn D, McCarthy C. All Aspects of Health Literacy Scale (AAHLS): developing a tool to measure functional, communicative and critical health literacy in primary healthcare settings.*Patient Educ Couns.*2013;90(2):247-53. doi:10.1016/j.pec.2012.10.019

4. Swearingen CJ, McCollum L, Daltroy LH, Pincus T, Dewalt DA, Davis TC. Screening for low literacy in a rheumatology setting: more than 10% of patients cannot read “cartilage,” “diagnosis,” “rheumatologist,” or “symptom.”*J Clin Rheumatol.*2010;16(8):359-64. doi:10.1097/RHU.0b013e3181fe8ab1

5. Cokely ET, Galesic M, Schulz E, Ghazal S, Garcia-Retamero R. Measuring risk literacy: the Berlin Numeracy Test. *Judgm Decis Mak*. 2012;7(1):25-47. doi:10.1017/S1930297500001819

6. Williams KP, Templin TN, Hines RD. Answering the call: a tool that measures functional breast cancer literacy.*J Health Commun.*2013;18(11):1310-25. doi:10.1080/10810730.2013.778367

7. Osborn CY, Davis TC, Bailey SC, Wolf MS. Health literacy in the context of HIV treatment: introducing the Brief Estimate of Health Knowledge and Action (BEHKA)-HIV version.*AIDS Behav.*2010;14(1):181-8. doi:0.1007/s10461-008-9484-z

8. Chew LD, Bradley KA, Boyko EJ. Brief questions to identify patients with inadequate health literacy.*Fam Med.*2004;36(8):588-94.

9. Haun J, Luther S, Dodd V, Donaldson P. Measurement variation across health literacy assessments: implications for assessment selection in research and practice.*J Health Commun.*2012;17 Suppl 3:141-59. doi:10.1080/10810730.2012.712615

10. Pleasant A, Maish C, O’Leary C, Carmona R. A theory-based self-report measure of health literacy: The Calgary Charter on Health Literacy scale. *Method Innov*. 2018;11(3). doi:10.1177/2059799118814394

11. Mazor KM, Rogers HJ, Williams AE, et al. The Cancer Message Literacy Tests: psychometric analyses and validity studies.*Patient Educ Couns.*2012;89(1):69-75. doi:10.1016/j.pec.2012.06.018

12. Williams KP, Templin TN. Bringing the real world to psychometric evaluation of cervical cancer literacy assessments with Black, Latina, and Arab women in real-world settings.*J Cancer Educ.*2013;28(4):738-43. doi:10.1007/s13187-013-0549-y

13. Austvoll-Dahlgren A, Guttersrud Ø, Nsangi A, Semakula D, Oxman AD. Measuring ability to assess claims about treatment effects: a latent trait analysis of items from the ‘Claim Evaluation Tools’ database using Rasch modelling.*BMJ Open.*2017;7(5):e013185. doi:10.1136/bmjopen-2016-013185

14. Luo H, Patil SP, Wu Q, et al. Validation of a combined health literacy and numeracy instrument for patients with type 2 diabetes.*Patient Educ Couns.*2018;101(10):1846-51. doi:10.1016/j.pec.2018.05.017

15. Samara J, Saunders L, Larson RF. Medical vocabulary knowledge among hospital patients. *J Health Hum Behav*. 2(2):83-92.

16. Curtis LM, Revelle W, Waite K, et al. Development and validation of the comprehensive health activities scale: a new approach to health literacy measurement.*J Health Commun.*2015;20(2):157-64. doi:10.1080/10810730.2014.917744

17. Weidmer BA, Brach C, Slaughter ME, Hays RD. Development of items to assess patients' health literacy experiences at hospitals for the Consumer Assessment of Healthcare Providers and Systems (CAHPS) Hospital Survey.*Med Care.*2012;50(9 Suppl 2):S12-21. doi:10.1097/MLR.0b013e31826524a0

18. Guttersrud O, Dalane JØ, Pettersen S. Improving measurement in nutrition literacy research using Rasch modelling: examining construct validity of stage-specific 'critical nutrition literacy' scales.*Public Health Nutr.*2014;17(4):877-83. doi:10.1017/S1368980013000530

19. Hanchate AD, Ash AS, Gazmararian JA, Wolf MS, Paasche-Orlow MK. The Demographic Assessment for Health Literacy (DAHL): a new tool for estimating associations between health literacy and outcomes in national surveys.*J Gen Intern Med.*2008;23(10):1561-6. doi:10.1007/s11606-008-0699-5

20. Davis TC, Wolf MS, Arnold CL, et al. Development and validation of the Rapid Estimate of Adolescent Literacy in Medicine (REALM-Teen): a tool to screen adolescents for below-grade reading in health care settings.*Pediatrics.*2006;118(6):e1707-14. doi:10.1542/peds.2006-1139

21. Mulvaney SA, Lilley JS, Cavanaugh KL, Pittel EJ, Rothman RL. Validation of the diabetes numeracy test with adolescents with type 1 diabetes.*J Health Commun.*2013;18(7):795-804. doi:10.1080/10810730.2012.757394

22. Huizinga MM, Elasy TA, Wallston KA, et al. Development and validation of the Diabetes Numeracy Test (DNT).*BMC Health Serv Res.*2008;8:96. doi:10.1186/1472-6963-8-96

23. Kayser L, Karnoe A, Furstrand D, et al. A multidimensional tool based on the eHealth Literacy Framework: development and initial validity testing of the eHealth Literacy Questionnaire (eHLQ).*J Med Internet Res.*2018;20(2):e36. doi:10.2196/jmir.8371

24. Norman CD, Skinner HA. eHEALS: The eHealth Literacy Scale.*J Med Internet Res.*2006;8(4):e27. doi:10.2196/jmir.8.4.e27

25. Rouquette A, Nadot T, Labitrie P, et al. Validity and measurement invariance across sex, age, and education level of the French short versions of the European Health Literacy Survey Questionnaire.*PLoS One.*2018;13(12):e0208091. doi:10.1371/journal.pone.0208091

26. Begley A, Paynter E, Dhaliwal SS. Evaluation tool development for food literacy programs.*Nutrients.*2018;10(11):1617. doi:10.3390/nu10111617

27. Doustmohammadian A, Omidvar N, Keshavarz-Mohammadi N, Abdollahi M, Amini M, Eini-Zinab H. Developing and validating a scale to measure Food and Nutrition Literacy (FNLIT) in elementary school children in Iran.*PLoS One.*2017;12(6):e0179196. doi:10.1371/journal.pone.0179196

28. Reynolds JS, Treu JA, Njike V, et al. The validation of a food label literacy questionnaire for elementary school children.*J Nutr Educ Behav.*2012;44(3):262-6. doi:10.1016/j.jneb.2011.09.006

29. Ownby RL, Acevedo A, Jacobs RJ, Caballero J, Waldrop-Valverde D. Quality of life, health status, and health service utilization related to a new measure of health literacy: FLIGHT/VIDAS.*Patient Educ Couns.*2014;96(3):404-10. doi:10.1016/j.pec.2014.05.005

30. Zhang XH, Thumboo J, Fong KY, Li SC. Development and validation of a functional health literacy test. *Patient*. 2009;2(3):169-178. doi:10.2165/11314850-000000000-00000

31. Osborn CY, Wallston KA, Shpigel A, Cavanaugh K, Kripalani S, Rothman RL. Development and validation of the General Health Numeracy Test (GHNT).*Patient Educ Couns.*2013;91(3):350-6. doi:10.1016/j.pec.2013.01.001

32. Galesic M, Garcia-Retamero R. Graph literacy: a cross-cultural comparison.*Med Decis Making.*2011;31(3):444-57. doi:[10.1177/0272989X10373805](https://doi.org/10.1177/0272989X10373805)

33. Rudd R, Kirsch I, Yamamoto K. Literacy and health in America. Policy information report (Report No. ED486416, 2004). Educational Testing Service. Accessed June 18, 2023. https://files.eric.ed.gov/fulltext/ED486416.pdf

34. Manganello JA, DeVellis RF, Davis TC, Schottler-Thal C. Development of the Health Literacy Assessment Scale for Adolescents (HAS-A).*J Commun Healthc.*2015;8(3):172-84. doi:10.1179/1753807615Y.0000000016

35. Abel T, Hofmann K, Ackermann S, Bucher S, Sakarya S. Health literacy among young adults: a short survey tool for public health and health promotion research.*Health Promot Int.*2015;30(3):725-35. doi:10.1093/heapro/dat096

36. Hahn EA, Choi SW, Griffith JW, Yost KJ, Baker DW. Health literacy assessment using talking touchscreen technology (Health LiTT): a new item response theory-based measure of health literacy.*J Health Commun.*2011;16 Suppl 3(Suppl 3):150-62. doi:10.1080/10810730.2011.605434

37. Paakkari O, Torppa M, Kannas L, Paakkari L. Subjective health literacy: Development of a brief instrument for school-aged children.*Scand J Public Health.*2016;44(8):751-7. doi:10.1177/1403494816669639

38. Jones K, Parker E, Mills H, Brennan D, Jamieson LM. Development and psychometric validation of a Health Literacy in Dentistry scale (HeLD).*Community Dent Health.*2014;31(1):37-43.

39. Tavousi M, Haeri-Mehrizi A, Rakhshani F, et al. Development and validation of a short and easy-to-use instrument for measuring health literacy: the Health Literacy Instrument for Adults (HELIA).*BMC Public Health.*2020;20(1):656. doi:10.1186/s12889-020-08787-2

40. Jordan JE, Buchbinder R, Briggs AM, et al. The health literacy management scale (HeLMS): a measure of an individual's capacity to seek, understand and use health information within the healthcare setting.*Patient Educ Couns.*2013;91(2):228-35. doi:10.1016/j.pec.2013.01.013

41. Ghanbari S, Ramezankhani A, Montazeri A, Mehrabi Y. Health Literacy Measure for Adolescents (HELMA): development and psychometric properties.*PLoS One.*2016;11(2):e0149202. doi:10.1371/journal.pone.0149202

42. Wu AD, Begoray DL, Macdonald M, et al. Developing and evaluating a relevant and feasible instrument for measuring health literacy of Canadian high school students.*Health Promot Int.*2010;25(4):444-52. doi:10.1093/heapro/daq032

43. Yuen E, Knight T, Dodson S, et al. Measuring cancer caregiver health literacy: validation of the Health Literacy of Caregivers Scale-Cancer (HLCS-C) in an Australian population.*Health Soc Care Community.*2018;26(3):330-44. doi:10.1111/hsc.12524

44. Osborne RH, Batterham RW, Elsworth GR, Hawkins M, Buchbinder R. The grounded psychometric development and initial validation of the Health Literacy Questionnaire (HLQ).*BMC Public Health.*2013;13:658. doi:10.1186/1471-2458-13-658

45. McCormack L, Bann C, Squiers L, et al. Measuring health literacy: a pilot study of a new skills-based instrument.*J Health Commun.*2010;15 Suppl 2:51-71. doi:10.1080/10810730.2010.499987

46. Bann CM, McCormack LA, Berkman ND, Squiers LB. The Health Literacy Skills Instrument: a 10-item short form.*J Health Commun.*2012;17 Suppl 3:191-202. doi:10.1080/10810730.2012.718042

47. Ko Y, Lee JY, Toh MP, Tang WE, Tan AS. Development and validation of a general health literacy test in Singapore.*Health Promot Int.*2012;27(1):45-51. doi:10.1093/heapro/dar020

48. Kim MT, Song HJ, Han HR, et al. Development and validation of the high blood pressure-focused health literacy scale.*Patient Educ Couns.*2012;87(2):165-70. doi:10.1016/j.pec.2011.09.005

49. Ownby RL, Waldrop-Valverde D, Hardigan P, Caballero J, Jacobs R, Acevedo A. Development and validation of a brief computer-administered HIV-Related Health Literacy Scale (HIV-HL).*AIDS Behav.*2013;17(2):710-8. doi:10.1007/s10461-012-0301-3

50. Mafutha NG, Mogotlane S, De Swardt H. Development of a hypertension health literacy assessment tool for use in primary healthcare clinics in South Africa, Gauteng.*Afr J Prim Health Care Fam Med.*2017;9(1):e1-e8. doi:10.4102/phcfm.v9i1.1305

51. Scior K, Furnham A. Development and validation of the Intellectual Disability Literacy Scale for assessment of knowledge, beliefs and attitudes to intellectual disability.*Res Dev Disabil.*2011;32(5):1530-41. doi:10.1016/j.ridd.2011.01.044

52. Rosaasen N, Taylor J, Blackburn D, Mainra R, Shoker A, Mansell H. Development and Validation of the Kidney Transplant Understanding Tool (K-TUT).*Transplant Direct.*2017;3(3):e132. doi:101097/TXD.0000000000000647

53. Nath CR, Sylvester ST, Yasek V, Gunel E. Development and validation of a literacy assessment tool for persons with diabetes.*Diabetes Educ.*2001;27(6):857-64. doi:10.1177/014572170102700611

54. Levin-Zamir D, Lemish D, Gofin R. Media Health Literacy (MHL): development and measurement of the concept among adolescents.*Health Educ Res.*2011;26(2):323-35. doi:10.1093/her/cyr007

55. Hanson-Divers EC. Developing a medical achievement reading test to evaluate patient literacy skills: a preliminary study.*J Health Care Poor Underserved.*1997;8(1):56-69. doi:10.1353/hpu.2010.0304

56. Schwartz LM, Woloshin S, Welch HG. Can patients interpret health information? An assessment of the medical data interpretation test.*Med Decis Making.*2005;25(3):290-300. doi:10.1177/0272989X05276860

57. Rawson KA, Gunstad J, Hughes J, et al. The METER: a brief, self-administered measure of health literacy.*J Gen Intern Med.*2010;25(1):67-71. doi:10.1007/s11606-009-1158-7

58. Sauceda JA, Loya AM, Sias JJ, Taylor T, Wiebe JS, Rivera JO. Medication literacy in Spanish and English: psychometric evaluation of a new assessment tool.*J Am Pharm Assoc (2003).*2012;52(6):e231-40. doi:10.1331/JAPhA.2012.11264

59. Weiss BD, Mays MZ, Martz W, et al. Quick assessment of literacy in primary care: the newest vital sign.*Ann Fam Med.*2005;3(6):514-22. doi:10.1370/afm.405

60. Rowlands G, Khazaezadeh N, Oteng-Ntim E, Seed P, Barr S, Weiss BD. Development and validation of a measure of health literacy in the UK: the newest vital sign.*BMC Public Health.*2013;13:116. doi:10.1186/1471-2458-13-116

61. Lipkus IM, Samsa G, Rimer BK. General performance on a numeracy scale among highly educated samples.*Med Decis Making.*2001;21(1):37-44. doi:10.1177/0272989X0102100105

62. Schapira MM, Walker CM, Miller T, et al. Development and validation of the Numeracy Understanding in Medicine Instrument short form.*J Health Commun.*2014;19 Suppl 2(0 2):240-53. doi:10.1080/10810730.2014.933916

63. Schapira MM, Walker CM, Cappaert KJ, et al. The numeracy understanding in medicine instrument: a measure of health numeracy developed using item response theory.*Med Decis Making.*2012;32(6):851-65. doi:10.1177/0272989X12447239

64. Gibbs HD, Ellerbeck EF, Gajewski B, Zhang C, Sullivan DK. The Nutrition Literacy Assessment Instrument is a valid and reliable measure of nutrition literacy in adults with chronic disease.*J Nutr Educ Behav.*2018;50(3):247-257.e1. doi:10.1016/j.jneb.2017.10.008

65. Gibbs HD, Harvey S, Owens S, Boyle D, Sullivan DK. Engaging experts and patients to refine the Nutrition Literacy Assessment Instrument.*BMC Nutr.*2017;3:71. doi:10.1186/s40795-017-0190-y

66. Diamond JJ. Development of a reliable and construct valid measure of nutritional literacy in adults.*Nutr J.*2007;6:5. doi:10.1186/1475-2891-6-5

67. Sabbahi DA, Lawrence HP, Limeback H, Rootman I. Development and evaluation of an oral health literacy instrument for adults.*Community Dent Oral Epidemiol.*2009;37(5):451-62. doi:10.1111/j.1600-0528.2009.00490.x

68. Kumar D, Sanders L, Perrin EM, et al. Parental understanding of infant health information: health literacy, numeracy, and the Parental Health Literacy Activities Test (PHLAT).*Acad Pediatr.*2010;10(5):309-16. doi:10.1016/j.acap.2010.06.007

69. Ayre J, Costa DSJ, McCaffery KJ, Nutbeam D, Muscat DM. Validation of an Australian parenting health literacy skills instrument: The parenting plus skills index.*Patient Educ Couns.*2020;103(6):1245-51. doi:10.1016/j.pec.2020.01.012

70. Manganello JA, Colvin KF, Chisolm DJ, Arnold C, Hancock J, Davis T. Validation of the Rapid Estimate for Adolescent Literacy in Medicine Short Form (REALM-TeenS).*Pediatrics.*2017;139(5):e20163286. doi:10.1542/peds.2016-3286

71. Richman JA, Lee JY, Rozier RG, Gong DA, Pahel BT, Vann WF. Evaluation of a word recognition instrument to test health literacy in dentistry: the REALD-99.*J Public Health Dent.*2007;67(2):99-104. doi:10.1111/j.1752-7325.2007.00022.x

72. Lee JY, Rozier RG, Lee SY, Bender D, Ruiz RE. Development of a word recognition instrument to test health literacy in dentistry: the REALD-30--a brief communication.*J Public Health Dent.*2007;67(2):94-8. doi: 10.1111/j.1752-7325.2007.00021.x

73. Erby LH, Roter D, Larson S, Cho J. The rapid estimate of adult literacy in genetics (REAL-G): a means to assess literacy deficits in the context of genetics.*Am J Med Genet A.*2008;146A(2):174-81. doi:10.1002/ajmg.a.32068

74. Bass PF, Wilson JF, Griffith CH. A shortened instrument for literacy screening.*J Gen Intern Med.*2003;18(12):1036-8. doi:10.1111/j.1525-1497.2003.10651.x

75. Davis TC, Crouch MA, Long SW, et al. Rapid assessment of literacy levels of adult primary care patients.*Fam Med.*1991;23(6):433-5.

76. Atchison KA, Gironda MW, Messadi D, Der-Martirosian C. Screening for oral health literacy in an urban dental clinic.*J Public Health Dent.*2010;70(4):269-75. doi:10.1111/j.1752-7325.2010.00181.x

77. Arozullah AM, Yarnold PR, Bennett CL, et al. Development and validation of a short-form, rapid estimate of adult literacy in medicine.*Med Care.*2007;45(11):1026-33. doi:10.1016/j.avsg.2008.10.005

78. Wallace LS, Ergen WF, Cassada DC, et al. Development and validation of the Rapid Estimate of Adult Literacy in Vascular Surgery (REAL_VS).*Ann Vasc Surg.*2009;23(4):446-52. doi:10.1016/j.avsg.2008.10.005

79. Lee SY, Stucky BD, Lee JY, Rozier RG, Bender DE. Short Assessment of Health Literacy-Spanish and English: a comparable test of health literacy for Spanish and English speakers.*Health Serv Res.*2010;45(4):1105-20. doi:10.1111/j.1475-6773.2010.01119.x

80. Duong TV, Aringazina A, Kayupova G, et al. Development and Validation of a New Short-Form Health Literacy Instrument (HLS-SF12) for the General Public in Six Asian Countries.*Health Lit Res Pract.*2019;3(2):e91-e102. doi:10.3928/24748307-20190225-01

81. McNaughton CD, Cavanaugh KL, Kripalani S, Rothman RL, Wallston KA. Validation of a short, 3-item version of the Subjective Numeracy Scale.*Med Decis Making.*2015;35(8):932-6. doi:10.1177/0272989X15581800

82. Morris NS, MacLean CD, Chew LD, Littenberg B. The Single Item Literacy Screener: evaluation of a brief instrument to identify limited reading ability.*BMC Fam Pract.*2006;7:21. doi:10.1186/1471-2296-7-21

83. Primack BA, Gold MA, Switzer GE, Hobbs R, Land SR, Fine MJ. Development and validation of a smoking media literacy scale for adolescents.*Arch Pediatr Adolesc Med.*2006;160(4):369-74. doi:10.1001/archpedi.160.4.369

84. Fagerlin A, Zikmund-Fisher BJ, Ubel PA, Jankovic A, Derry HA, Smith DM. Measuring numeracy without a math test: development of the Subjective Numeracy Scale.*Med Decis Making.*2007;27(5):672-80. doi:10.1177/0272989X07304449

85. Wang J, Thombs BD, Schmid MR. The Swiss Health Literacy Survey: development and psychometric properties of a multidimensional instrument to assess competencies for health.*Health Expect.*2014;17(3):396-417. doi:10.1111/j.1369-7625.2012.00766.x

86. Gong DA, Lee JY, Rozier RG, Pahel BT, Richman JA, Vann WF. Development and testing of the Test of Functional Health Literacy in Dentistry (TOFHLiD).*J Public Health Dent.*2007;67(2):105-12. doi:10.1111/j.1752-7325.2007.00023.x

87. Schwartz LM, Woloshin S, Black WC, Welch HG. The role of numeracy in understanding the benefit of screening mammography.*Ann Intern Med.*1997;127(11):966-72. doi:10.7326/0003-4819-127-11-199712010-00003

88. Hyde LL, Boyes AW, Evans TJ, Mackenzie LJ, Sanson-Fisher R. Three-factor structure of the eHealth Literacy Scale among magnetic resonance imaging and computed tomography outpatients: a confirmatory factor analysis.*JMIR Hum Factors.*2018;5(1):e6. doi:10.2196/humanfactors.9039

89. Stucky BD, Lee JY, Lee SY, Rozier RG. Development of the two-stage rapid estimate of adult literacy in dentistry.*Community Dent Oral Epidemiol.*2011;39(5):474-80. doi:10.1111/j.1600-0528.2011.00619.x

90. Irvin VL, Rohlman D, Vaughan A, Amantia R, Berlin C, Kile ML. Development and validation of an environmental health literacy assessment screening tool for domestic well owners: the Water Environmental Literacy Level Scale (WELLS).*Int J Environ Res Public Health.*2019;16(5):881. doi:10.3390/ijerph16050881

91. Wang ML, Little TV, Frisard C, Borg A, Lemon SC, Rosal MC. Development and validation of a Weight Literacy Scale in English and Spanish.*PLoS One.*2018;13(10):e0204678. doi:10.1371/journal.pone.0204678
